# Domain-Aware Few-Shot Learning for Optical Coherence Tomography Noise Reduction

**DOI:** 10.3390/jimaging9110237

**Published:** 2023-10-30

**Authors:** Deborah Pereg

**Affiliations:** 1 Department of Mechanical Engineering, Massachusetts Institute of Technology, Cambridge, MA 02139, USA; deborahp@mit.edu; 2 School of Engineering and Applied Sciences, Harvard University, Cambridge, MA 02138, USA

**Keywords:** few-shot learning, image denoising, inverse problems, speckle suppression

## Abstract

Speckle noise has long been an extensively studied problem in medical imaging. In recent years, there have been significant advances in leveraging deep learning methods for noise reduction. Nevertheless, adaptation of supervised learning models to unseen domains remains a challenging problem. Specifically, deep neural networks (DNNs) trained for computational imaging tasks are vulnerable to changes in the acquisition system’s physical parameters, such as: sampling space, resolution, and contrast. Even within the same acquisition system, performance degrades across datasets of different biological tissues. In this work, we propose a few-shot supervised learning framework for optical coherence tomography (OCT) noise reduction, that offers high-speed training (of the order of seconds) and requires only a single image, or part of an image, and a corresponding speckle-suppressed ground truth, for training. Furthermore, we formulate the domain shift problem for OCT diverse imaging systems and prove that the output resolution of a despeckling trained model is determined by the source domain resolution. We also provide possible remedies. We propose different practical implementations of our approach, verify and compare their applicability, robustness, and computational efficiency. Our results demonstrate the potential to improve sample complexity, generalization, and time efficiency, for coherent and non-coherent noise reduction via supervised learning models, that can also be leveraged for other real-time computer vision applications.

## 1. Introduction

OCT employs low-coherence interferometry to produce cross-sectional tomographic images of the internal structure of biological tissues [1,2]. It is routinely used for diagnostic imaging, primarily of the retina and coronary arteries [3]. The axial resolution obtainable is in the range 2 to 15 µm [4], with a depth range of around 1–2 mm. Unfortunately, OCT images are often degraded by speckle noise [5,6], creating apparent grain-like structures in the image, with a size as large as the spatial resolution of the OCT system. Speckle noise significantly degrades images and complicates interpretation and medical diagnosis by confounding tissue anatomy and masking changes in tissue scattering properties.

Speckle suppression is often achieved by incoherent averaging of images with different speckle realizations [7], e.g., through angular compounding [8,9]. Averaging methods attempt to preserve the resolution while suppressing speckle arising from non-resolved tissue structure; yet, some methods produce blurred images. Moreover, although effective at suppressing speckle in ex vivo tissues or in preclinical animal research, the additional time and data throughput required to obtain multiple speckle realizations can often make this approach incompatible with clinical in vivo imaging.

Consequently, many numerical algorithms attempt to computationally suppress speckle, to name a few: non-linear filtering [10], non-local means (NLMs) [11,12], and block matching and 3D filtering (BM3D) [13]. The majority of these algorithms employ an image denoiser treating speckle as independent and identically distributed (i.i.d) Gaussian noise. The solution can sometimes be sensitive to parameters’ fine-tuning. Some algorithms also rely on accurately registered volumetric data, which is challenging to obtain in clinical settings.

Recently, the speckle reduction task has been extensively investigated from a supervised learning perspective [14,15,16]. As is known, most supervised learning data-driven methods require a large training dataset. In OCT, Dong et al. (2020) [17] trained a super-resolution generative adversarial network (SRGAN) [18,19] with hardware-based speckle-suppressed ex vivo samples defining the ground truth. Namely, they used 200,000 speckle-modulating OCT images of size 800×600 for training. Chintada et al. (2023) [20] used a conditional-GAN (cGan) [21] trained with hundreds of retinal data B-scans, with NLM [12] as ground truth. Ma et al. (2018) [22] also used a cGAN to perform speckle reduction and contrast enhancement for retinal OCT images by adding an edge loss function to the final objective. Clean images for training were obtained by averaging B-scans from multiple OCT volumes.

That said, there has been a growing amount of evidence demonstrating that supervised learning methods, specifically in the context of computational imaging and inverse problems, may require significantly smaller datasets. For example, it was observed that, for image restoration for florescence microscopy [23], even a small number of training images led to an acceptable image restoration quality (e.g., 200 patches of size 64×64×16). Pereg et al. (2020) [24] used a single simplified synthetic image example for seismic inversion. Several works have explored the use of few-shot learning for transfer learning [25,26]. For example, Huang et al. (2022) employed a recurrent neural net (RNN) for few-shot transfer learning of holographic image reconstruction [25]. The RNN was first trained with ∼2000 unique training images of three sample types, then its parameters remained fixed as a backbone model. The transfer learning phase required only 80 examples. Some progress has been made for few-shot learning for medical imaging primarily for classification and segmentation [27,28]. To our knowledge, our work presented here is the first research work addressing few-shot learning for OCT noise reduction.

In learning theory, domain shift is a change of the data distribution between the source domain (training dataset) and target domain (the test dataset). Despite advances in data augmentation and transfer learning, neural networks often fail to adapt to unseen domains. For example, convolutional neural networks (CNNs) trained for segmentation tasks can be highly sensitive to changes in resolution and contrast. Performance often degrades even within the same imaging modality. A general review of domain adaptation (DA) for medical image analysis can be found in [29]. The different approaches are separated into shallow and deep DA models, further divided into supervised, semi-supervised, and unsupervised DA, depending on the availability of labeled data in the target domain. Generally speaking, the appropriate DA approach depends on the background and the properties of the specific problem. Many DA methods suggest ways to map the source and target domains to a shared latent space, whereas generative DA methods attempt to translate the source to the target or vice versa. In our study, we focused on a simple, yet efficient physics-aware unsupervised DA approach for the case of a change in the OCT imaging system. Namely, only unlabeled data are available for the target domain. This problem is also referred to in the literature as domain generalization [30], and it has been hardly explored in medical imaging so far [31].

Our aim in this work is to investigate few-shot learning as an efficient tool for OCT speckle reduction with limited ground truth training data. To this end, we first prove that the output resolution of a supervised learning speckle-suppression system is determined by the sampling space and the resolution of the source acquisition system. We also mathematically define the effects of the domain shift on the target output image. In light of the theoretical analysis, we promote the use of a patch-based learning approach. We propose a recurrent neural net (RNN) framework to demonstrate the applicability and efficiency for few-shot learning for OCT speckle suppression. We demonstrate the use of a single-image training dataset that generalizes well. The proposed approach introduces a significant decrease in the training time and required computational resources. Training takes about *2–25 seconds* on a GPU workstation and a few minutes on a CPU workstation (2–4 minutes). We further propose novel upgrades for the original RNN framework and compare their performance. Namely, we introduce a one-shot patch-based RNN-mini-GAN architecture. We further demonstrate the increased SNR achieved via averaging overlapping patches. Furthermore, we recast the speckle-suppression network as a deblurring system. We further propose a patch-based one-shot learning U-Net [32] and compare its results with the three different RNN models’ results. We illuminate the speckle reduction dependence of the acquisition system, via known lateral and axial sampling space and resolution, and offer simple strategies for training and testing under different acquisition systems. Finally, our approach can be applicable to other learning architectures, as well as other applications where the signal can be processed locally, such as speech and audio, video, seismic imaging, MRI, ultrasound, natural language processing, and more. The results in this paper are a substantial extension that replaces our non-published previous work ([33], Section 6.2).

## 2. Preliminaries

### 2.1. Speckle Statistics

OCT tomograms display the intensity of the scattered light, as the log-valued squared norm of the complex-valued tomogram. It is assumed that the contributions from structural features beyond the imaging resolution of OCT add up coherently and generate a random speckle pattern [5,6]. Speckle is not an additive statistically independent noise, but rather, unresolved spatial information originating in the interference of many sub-resolution spaced scatterers [34]. Speckle also plays an important role in other fields, e.g., synthetic-aperture radar and ultrasound medical imaging. Exact analogs of the speckle phenomenon appear in many other fields and applications. The squared magnitude of the finite-time Fourier transform (FFT) (the periodogram) of a sampled function of almost any random process shows fluctuations in the frequency domain that have the same single-point (pixel) statistics as speckle [35]. Generally speaking, speckle appears in a signal when the signal is a linear combination of independently random phased additive complex elements. The resulting sum is a random walk, which may exhibit constructive or destructive interference depending on the relative phases. The intensity of the observed wave is the squared norm of this sum.

As mention above, speckle in OCT arises from sub-resolution reflectors. In addition, optical frequency domain imaging (OFDI) OCT is the FFT of its measured spectral components and, therefore, exhibits noise typical to any periodogram. When a pixel’s value is a result of the sum of a large enough number of reflectors, the sum, according to the central limit theorem, has a Gaussian distribution. In this case, assuming a uniform phase, for fully developed speckle, the intensity is distributed according to an exponential probability density:

(1) $$p_{Y|X}\left(y|x\right)=\frac{1}{x}\exp\left(-\frac{y}{x}\right),\;y>0$$

where *y* is the measured intensity pixel value and *x* is the mean intensity, defining the ground truth. In other words, the fluctuations of fully developed speckle are of the same order as the the ground truth pixel value, which renders this type of noise particularly destructive and visually disturbing. Speckle that is not fully developed would have a more-complicated distribution formulation, depending on the number of phasors and their amplitudes’ and phases’ distribution.

### 2.2. RNN Encoder–Decoder Framework

Assume an observation data sequence $y={\left[y_{0},y_{1},\ldots ,y_{L_{t-1}}\right]}^{T}$, $y_{t}\in R^{N\times 1}$, $t\in [0,L_{t}-1]$, and a corresponding output sequence $x={\left[x_{0},x_{1},\ldots ,x_{L_{t}-1}\right]}^{T}$, $x_{t}\in R^{P\times 1}$. Superscript ${}^{T}$ denotes the transpose operation. The RNN forms a map f:y→z, from the input data to the latent space variables. That is, for input $y_{t}$ and state $z_{t}$ at time step *t*, the RNN output is generally formulated as $z_{t}=f\left(z_{t-1},y_{t}\right)$ [36]. Hereafter, we focus on the specific parametrization:

(2) $$z_{t}=\sigma \left(W_{zy}^{T}y_{t}+W_{zz}^{T}z_{t-1}+b\right),$$

where σ is an activation function, $W_{zy}\in R^{N\times n_{n}}$ and $W_{zz}\in R^{n_{n}\times n_{n}}$ are weight matrices, and $b\in R^{n_{n}\times 1}$ is the bias vector. At t=0, the previous outputs are zero. Here, we used the ReLU activation function, ReLU(z)=max{0,z}. We wrapped each cell with a fully connected layer with the desired final output $x_{t}\in R^{P\times 1}$, such that $x_{t}=\mathrm{FC}\left(z_{t}\right)$.

Traditionally, RNNs are used for processing of time-related signals, to predict future outcomes, and for natural language processing tasks such as handwriting recognition [37] and speech recognition [38]. In computer vision, recurrent convolutional networks (RCNNs) have been proposed for object recognition [39]. Pixel-RNN [40] sequentially predicts pixels in an image along the two spatial dimensions.

## 3. Domain-Aware Speckle Suppression

Let us denote f(z,x)∈C as the ground truth ideal tomogram perfectly describing the depth sample reflectivity. Here, $\left\{\left(z,x\right):z,x\geq 0,\left(z,x\right)\in R^{2}\right\}$ are continuous axial and lateral spatial axes. A measured tomogram can be formulated as

(3) $$Y\left(z,x\right)=10\log_{10}\left({\left|f\left(z,x\right)*\alpha \left(z,x\right)\right|}^{2}\right).$$

where ∗ denotes the convolution operation and α(z,x) is a point spread function (PSF). In the discrete setting, assuming $F_{s}^{z}$, $F_{s}^{x}$ are the axial and lateral sampling rates, respectively, and that the set of measured values at $\left\{z_{m},x_{n}\right\}$ lie on the grid $m/F_{s}^{z}$ and $n/F_{s}^{x}$, m,n∈N,

(4) $$Y\left[m,n\right]=10\log_{10}\left(|f\left[m,n\right]*\alpha \left[m,n\right]{|}^{2}\right).$$

**Figure 1:**
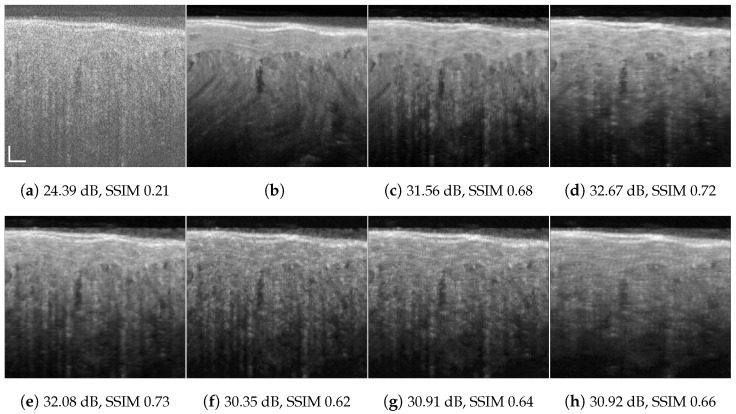
Chicken muscle speckle suppression results: (**a**) speckled acquired tomogram $p_{x}=3$; (**b**) ground truth averaged over 901 tomograms; (**c**) OCT-RNN trained with 100 first columns of chicken muscle; (**d**) RNN-GAN trained with 100 first columns of chicken muscle and blueberry, $p_{x}^{s}=p_{x}^{t}=3$; (**e**) RNN-GAN trained with 200 columns of chicken decimated by a factor 8/3 in the lateral direction, $p_{x}^{s}=1$. System and tissue mismatch: (**f**) DRNN trained with 100 columns of human retinal image, $p_{x}^{s}=2$; (**g**) DRNN following lateral decimation of the target input by a factor of 4/3, $p_{x}^{s}=p_{x}^{t}=2$; (**h**) DRNN following lateral decimation of the target input by 8/3, $p_{x}^{t}=1$. Scale bars are 200 µm.

A speckle-suppressed tomogram can be viewed as the incoherent mean of coherent tomograms with different speckle realizations [6,41] (see e.g., Figure 1):

(5) $$X\left[m,n\right]=10\log_{10}\left(|f\left[m,n\right]{|}^{2}*|\alpha \left[m,n\right]{|}^{2}\right).$$

In OCT (using a wavelength-swept source (SS-OCT) or Fourier domain/spectral domain (FD/SD-OCT)) [42,43], the axial direction corresponds to the depth at a certain scan location of the imaged sample. The axial imaging range $\Delta_{z}$ is given by the central wavelength and the wavelength sampling. The axial sampling space is $\delta z=\frac{\Delta_{z}}{n_{z}}$, where $n_{z}$ is the total A-line number of pixels. In the axial direction, the PSF effective width $\omega_{z}$ is determined by the FFT of a zero-padded Hanning window. The lateral direction corresponds to the direction of image scanning, such that assembling all A-lines of a lateral scan into a B-scan forms a cross-sectional image. In the lateral direction, the PSF has a Gaussian shape proportional to $\exp(-2x^{2}/w_{x}^{2})$, where $w_{x}$ is referred to as the waist. δx is the lateral sampling space. Therefore, α[m,n] is separable and can be expressed as $\alpha \left[m,n\right]=\alpha_{x}\left[m\right]*\alpha_{z}\left[n\right]$. Note that the resolution and sampling rate are known parameters of an OCT imaging system.

In matrix–vector form, we denote an input (log-scaled) image $Y\in R^{L_{r}\times J}$ that is a corrupted version of $X\in R^{L_{r}\times J}$, such that Y=X+N, where $N\in R^{L_{r}\times J}$ is an additional noise term. Note that, for the case of image despeckling, we do not assume that the entries of N are either i.i.d. or that it is uncorrelated with X. Our task is to recover X. That is, we attempt to find an estimate $\hat{X}$ of the unknown ground truth X.

Let us assume a source training set $D_{S}=\left\{{\left\{y_{i},x_{i}\right\}}_{i=1}^{m}:y_{i}\in R^{L_{t}\times N_{x}},x_{i}\in R^{L_{t}\times N_{x}}\right\}$, where $\left\{y_{i},x_{i}\right\}∼P_{S}$ are image patches sampled from a source domain S as the ground truth. The learning system is trained to output a prediction rule $F_{S}:Y\to X$. We assume an algorithm that trains the predictor by minimizing the training error (empirical error or empirical risk). The domain shift problem assumes a target domain T with samples from a different distribution $\left\{y_{i},x_{i}\right\}∼P_{T}$.

**Assumption** **1** (Speckle Local Ergodicity)**.** *Denote $y_{i}$ as a patch centered around pixel i of the image Y. $P_{Y}\left(y_{i}\right)$ is the probability density of a patch $y_{i}$. Under the assumption that pixels in close proximity are a result of shared similar sub-resolution scatterers, we assume ergodicity of the Markov random field (MRF) (e.g., [44]) of patches $y_{i}$ consisting of pixels in close proximity.*

In other words, the probability distribution of a group of pixels’ values in close spatial proximity is defined by the same density across the entire image. This assumption takes into account that some of these patches correspond to fully developed speckle, non-fully developed speckle, and a combination of both. Note that the measured pixels’ values are correlated. That said, this assumption could be somewhat controversial, particularly in surroundings of abrupt changes in the signal intensity. However, since our images tend to have a layered structure and the PSF visible range is about 7–9 pixels in each direction, we will make this assumption.

**Definition** **1** (Sampling resolution ratio)**.** *We define the lateral sampling resolution ratio $p_{x}≜\left[\frac{\omega_{x}}{\delta x}\right]$ in pixels and the axial sampling resolution ratio as $p_{z}≜\left[\frac{\omega_{z}}{\delta z}\right]$, where [·] denotes rounding to the closest integer. That is, in a discrete setting, $p_{z}$ and $p_{x}$ are the number of pixels capturing the effective area of the PSF in each direction. The superscripts ${}^{t}$ and ${}^{s}$ denote the target and source, respectively.*

**Theorem** **1** (Domain-Shift Speckle Suppression Theorem)**.** *A learned patch-based speckle suppression mapping $F_{S}:y^{s}\to x^{s}$ does not require domain adaptation. However, the output ${\hat{x}}^{t}=F_{S}\left\{y^{t}\right\}$ resolution will be determined by the source domain resolution. Mathematically, denote $\alpha^{s}\left[m,n\right]$, $\alpha^{t}\left[m,n\right]$ as the discrete PSF in the source and target domain, respectively, such that*

(6) $$\alpha^{s}\left[m,n\right]*\alpha^{s\to t}\left[m,n\right]=\alpha^{t}\left[m,n\right]*\alpha^{t\to s}\left[m,n\right],$$

*where $\alpha^{s\to t}\left[m,n\right]$ and $\alpha^{t\to s}\left[m,n\right]$ are complementary impulse responses leading from one domain to the other. When applying the trained system to the target input, we have*

(7) $$\begin{array}{cc} & {\hat{x}}^{t}\left[m,n\right]=F_{S}\left(y^{t}\left[m,n\right]\right)=x^{s}\left[m,n\right]{|}_{y^{s}\left[m,n\right]=y^{t}\left[m,n\right]}\\ & =|f^{s}\left[m,n\right]{|}^{2}*|\alpha^{s}\left[m,n\right]{|}^{2}{|}_{f^{t}\left[m,n\right]*\alpha^{s\to t}\left[m,n\right]=f^{s}\left[m,n\right]*\alpha^{t\to s}\left[m,n\right]}. \end{array}$$

We refer the reader to Appendix A for the proof and a detailed explanation. For example, if $p_{z}^{s}=p_{z}^{t}$ and $p_{x}^{s}<p_{x}^{t}$, there exist $\alpha_{x}^{s\to t}\left[n\right]$ such that $\alpha_{x}^{t}\left[n\right]=\alpha_{x}^{s}\left[n\right]*\alpha_{x}^{s\to t}\left[n\right]$. Then, we have

$$\begin{array}{cc} {\hat{x}}^{t}\left[m,n\right] & =F_{S}\left(y^{t}\left[m,n\right]\right)=\\ & =|f^{t}\left[m,n\right]*\alpha^{s\to t}\left[m,n\right]{|}^{2}*|\alpha^{s}\left[m,n\right]{|}^{2}. \end{array}$$

In other words, the output resolution is determined by the source resolution $\alpha^{s}\left[m,n\right]$. The tomogram component $|f^{t}\left[m,n\right]*\alpha^{s\to t}\left[m,n\right]|$ is a low-resolution version of the target tomogram $|f^{t}\left[m,n\right]|$:

1. If $p_{x}^{s}<p_{x}^{t}$ or $p_{z}^{s}<p_{z}^{t}$, then the system’s prediction for an input in the target domain may have additional details or artificially enhanced resolution details, which would not naturally occur with other denoising mechanisms. Examples illustrating this phenomena are illustrated in Figure 1e–f. Possible remedies: train with a larger analysis patch size, longer training, and upsampling (interpolation) of source images (or decimation of target images).
2. If $p_{x}^{s}>p_{x}^{t}$ or $p_{z}^{s}>p_{z}^{t}$, then the network’s output is blurred in the corresponding direction (e.g., Figure 1h). Possible remedies: train with a smaller analysis patch size, downsample (decimate) the training image (or upsample target images). In this case, the target has details that are smaller (in pixels) than the minimal speckle size of the source, which could be interpreted by the trained predictor as noise; thus, the trained predictor may simply smear them out.

Any combination of relations $p_{z,x}^{s}≶p_{z,x}^{t}$ along the different image axes is possible. For our OCT data, the resolution ratio mostly differs in the lateral direction (see Table 1). Note that, for some OCT systems, the sampling space is below the Nyquist rate. The preprocessing domain adaptation stage can be applied either to the source data or the target data, interchangeably, depending on the desired target resolution. We note that (6) does not apply to any general pair of PSFs, but in our case study, it is safe to assume that there exist $\alpha^{s\to t}\left[m,n\right]$ and $\alpha^{t\to s}\left[m,n\right]$ that approximately satisfy (6).

For simplicity, we assumed a spatially invariant model that does not take into consideration light–matter interactions, optical scattering, and attenuation. It may also be argued that this model is not unique to OCT and could be applied to other modalities. Nevertheless, in practice, the proposed approach is effective and yields perceptually improved results. The above analysis is not restricted to OCT and can be easily modified and applied to other degradation processes and other applications.

## 4. Patch-Based Few-Shot Learning

**Figure 2:**
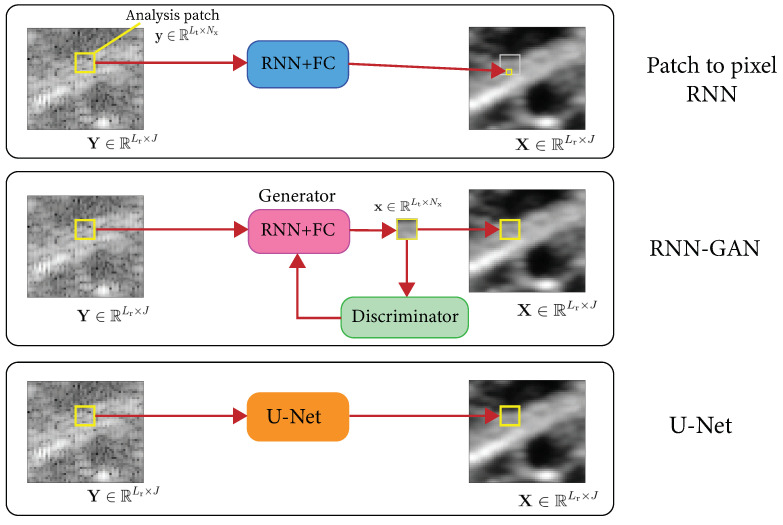
Proposed RNN, RNN-GAN, and U-Net schematic.

The initial RNN setting described in this subsection has been previously employed for seismic imaging [24,45,46]. Hereafter, the mathematical formulation focuses on the settings of the OCT despeckling task. Nonetheless, the model can be applied to a wide range of applications. We emphasize the potential of this framework and expand and elaborate its application, while connecting it to the theoretical intuition in Theorem 1. We also propose possible upgrades that further enhance the results in our case study, as shown in Figure 2.

Most OCT images have a layered structure and exhibit strong relations along the axial and lateral axes. RNNs can efficiently capture those relations and exploit them. That said, as demonstrated below, the proposed framework is not restricted to images that exhibit a layered structure nor to the specific RNN-based encoder–decoder architecture.

**Definition** **2** (Analysis patch [46])**.** *We define an analysis patch as a 2D patch of size $L_{t}\times N_{x}$ enclosing $L_{t}$ time (depth) samples of $N_{x}$ consecutive neighboring columns of the observed image $Y\in R^{L_{r}\times J}$. Assume $\left\{n_{L},n_{R}\in N:n_{L}+n_{R}=N_{x}-1\right\}$. Then, the analysis patch associated with a point at location (i,j) is defined by*

$$A_{k,l}^{\left(i,j\right)}=\left\{Y\left[i+k,j+l\right]:k,l\in Z,1-L_{t}\leq k\leq 0,\;-n_{L}\leq l\leq n_{R}\right\}.$$

An analysis patch $A^{\left(i,j\right)}\in R^{L_{t}\times N_{x}}$ is associated with a pixel X[i,j] in the output image. To produce a point in the estimated $\hat{X}\left[i,j\right]$, we set an input to the RNN as an analysis patch, i.e., $y=A^{\left(i,j\right)}$. Each time step input is a group of $N_{x}$ neighboring pixels of the same corresponding time (depth). In other words, in our application, $n_{i}=N_{x}$ and $y_{t}={\left[Y\left[t,j-n_{L}\right],\ldots ,Y\left[t,j+n_{R}\right]\right]}^{T}$. We set the size of the output vector $z_{t}$ to one expected pixel (P=1), such that x is expected to be the corresponding reflectivity segment, $x={\left[X\left[i-\left(L_{t}-1\right),j\right],\ldots ,X\left[i,j\right]\right]}^{T}$. Lastly, we ignore the first $L_{t}-1$ values of the output x, and set the predicted reflectivity pixel $\hat{X}\left[i,j\right]$ as the last one, i.e., $x_{L_{t}}$. The analysis patch moves across the image and produces all predicted points in the same manner. Each analysis patch and a corresponding output segment (or patch) are an instance for the net. The size and shape of the analysis patch define the geometrical distribution of data samples for inference.

### 4.1. Despeckling Reformulated as Image Deblurring

Despite the low-frequency bias of over-parametrized DNNs [47], previous works [46] demonstrated the ability of the proposed framework to promote high frequencies and super-resolution. To explore this possibility, we recast the framework described above as a deblurring task. This is achieved simply by applying a low-pass filter to the input speckled image and, then, training the system to deblur the image. Namely, given a noisy image Y, the analysis patches is extracted from the input image $\hat{Y}=\mathrm{HY}$, where H is a convolution matrix of a 2D low-pass filter. We will refer to this denoiser as deblurring RNN (DRNN).

### 4.2. Averaging Patches

**Figure 3:**
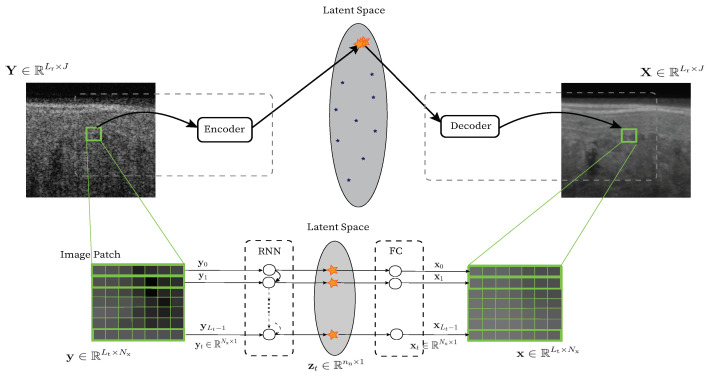
Illustration of the proposed patch-to-patch RNN encoder–decoder.

Given a noisy image Y, an alternative approach is to decompose it into overlapping patches, denoise every patch separately, and finally, combine the results by simple averaging. This approach of averaging of overlapping patch estimates is common in patch-based algorithms [48,49], such as the expected patch log-likelihood (EPLL) [50]. It also improves the SNR since we are averaging for every pixel a set of different estimates. Mathematically speaking, the input analysis patch is still $y=A^{\left(i,j\right)}\in R^{L_{t}\times N_{x}}$. However, in this configuration, the output is no longer a 1D segment, but a corresponding output 2D patch. In other words, $n_{i}=N_{x}$, $x_{t}={\left[X\left[t,j-n_{L}\right],\ldots ,X\left[t,j+n_{R}\right]\right]}^{T}$ such that $P=N_{x}$ (see Figure 3).

### 4.3. Incremental Generative Adversarial Network

Image restoration algorithms are typically evaluated by some distortion measure (e.g., PSNR, SSIM) or by human opinion scores that quantify the perceived perceptual quality. It has long been established that distortion and perceptual quality are at odds with each other [51]. As mentioned above, previous works adopt a two-stage training [17,18]. The first stage trains the generator with a content loss, while, in the second stage, initialized by the generator’s pre-trained weights, we train both a generator *G* and a discriminator *D*. Therefore, we propose adding a second stage of training with a combined MSE and adversarial loss:

(8) $$L_{G}=L_{\mathrm{MSE}}+\lambda L_{\mathrm{ADV}},$$

where λ is a constant balancing the losses. The generator *G* remains a patch-to-patch RNN-based predictor (with or without averaging patches). To this end, we design and showcase a patch discriminator of extremely low complexity, which consists simply of two fully connected layers. We will refer to this approach as RNN-GAN.

The above framework could be generalized to 3D images $X_{3D}\in R^{L_{r}\times J_{x}\times J_{y}}$ using a 3D analysis volume of size $L_{t}\times N_{x}\times N_{y}$. The analysis volume is then defined by $N_{x}$ and $N_{y}$, the number of A-lines and B-scans taken into account along the lateral axes, and $L_{t}$ depth samples along the axial axis. It can be defined to associate with a point in its center or in an asymmetrical manner. In a similar manner to the 2D configuration, for each output voxel, the analysis volume would be an instance input to the RNN. Moving the analysis volume along the 3D observation image produces the entire 3D predicted despeckled volume.

The underlying assumption of the proposed approach is that the mapping from each input patch to an output point or patch is statistically unchanging. That is, the data is stationary. In practice, this assumption is controversial and does not always hold. Yet, assuming spatial invariance is helpful for introducing the major processes affecting the image quality into the model and is standard in the image-processing literature. As presented in Section 5, in practice, this simplification does not necessarily lead to degraded results in comparison with the despeckled ground truth. The learned mapping is able to effectively capture the imaging degradation process despite its inherent statistical complexity.

### 4.4. Few-Shot U-Net

As is known, a U-Net is a convolutional neural network that was developed for biomedical image segmentation [32] and achieved state-of-the-art results in numerous applications. One of the U-Net’s advantages is that it is flexible with respect to its input size. Inspired by the above approach, we further propose a patch-based one-shot learning U-Net. In other words, the U-Net is trained with random patches cropped from a *single* input–output pair (or a few images). Then, the U-Net is applied to a larger image as desired by the user.

## 5. Experimental Results

Here, we show examples of our proposed few-shot domain-aware supervised learning despeckling approach with OCT experimental data, for demonstration. We investigated three one-shot learning challenging cases: (1) matching tissue and matching acquisition systems, where we used one image or part of an image for training and other images of the same tissue acquired by the same system for testing; (2) tissue type mismatch; (3) tissue type and acquisition system mismatch. Table 1 presents the acquisition parameters, namely axial and lateral sampling spaces in tissue, $N_{H}$—the effective number of measured spectral points vs. $N_{\mathrm{FFT}}$—the total number of FFT points after zero padding, $\omega_{x}$—the waist in µm, axial and lateral sampling resolution ratios in pixels, and the cropped region of interest (ROI) image sizes.

For all experiments, we set the number of neurons as $n_{n}=1000$. Increasing the number of neurons did not improve the results significantly, but increases training time. The analysis patch size is [15,15]. Patch size can affect the results’ higher frequencies. Larger patches create frequency bias in favor of lower frequencies. For the DRNN we used a Gaussian filter of size [7,7] and standard deviation σ=1. For the RNN-GAN we employed overlapping patches averaging to promote additional noise reduction. As mentioned above, our discriminator consists solely of 2 fully-connected layers. At the second adversarial stage the generator’s loss was modified to include a content loss term and an adversarial loss term $L_{G}=L_{\mathrm{MSE}}+\lambda L_{\mathrm{ADV}}$. We used the Adam-optimizer [52] with $\beta_{1}$ = 0.5, $\beta_{2}$ = 0.9. The initial learning rate is $10^{-4}$.

**Table 1:** Acquisition system parameters.

	Chicken and Blueberry	Chicken Skin	Cucumber	Retina	Cardiovascular-I [43]	Cardiovascular-II [53]
δz (µm)	6	4.78	4.78	3.75	4.84	4.43
$N_{H}/N_{\mathrm{FFT}}$	1600/2048	844/1024	844/1024	1024/2048	768/1024	800/1024
$p_{z}$	3	3	3	3	3	3
δx (µm)	3.06	2.5	8	9	∼12.2	∼24.4
$\omega_{x}$ (µm)	8.28	4.14	8.28	18	30	30
$p_{x}$	3	2	1	2	2	1
ROI image size	350×700, 380×944	320×650	420×401	448×832	1024×1024	1024×1024

*Ex Vivo OCT Samples*

As the ground truth for training and testing, we used hardware-based speckle mitigation obtained by dense angular compounding, in a method similar to [8]. That is, ground truth images for chicken muscle, blueberry, chicken skin, and cucumber sample tissues, as presented in Figure 1b and Figures 6–8b, were acquired by an angular compounding (AC) system using sample tilting in combination with a model-based affine transformation to generate speckle-suppressed ground truth data [54]. Note that AC via sample tilting is not possible for in vivo samples.

*Retinal Data*

We used retinal data acquired by a retinal imaging system similar to [55]. As the ground truth for training and testing, we used NLM-based speckle-suppressed images [12]. Note that the NLM is considered relatively slow (about 23 s for a B-scan of size 1024×1024). Images were cropped to a size of 448×832.

*Cardiovascular OCT*

Finally, we tested our trained systems with OCT data of coronary arteries acquired with two imaging systems. For these datasets, we have no ground truth available. The first dataset, referred to as Cardiovascular-I [43], was acquire with in-house built catheters, for human cadaver imaging. The second human-heart coronary dataset, Cardiovascular-II [53], was acquired with a second clinical system, where there is usually a guidewire in place. Since imaging time is critical, only 1024 A-lines per rotation were acquired.

**Figure 4:**
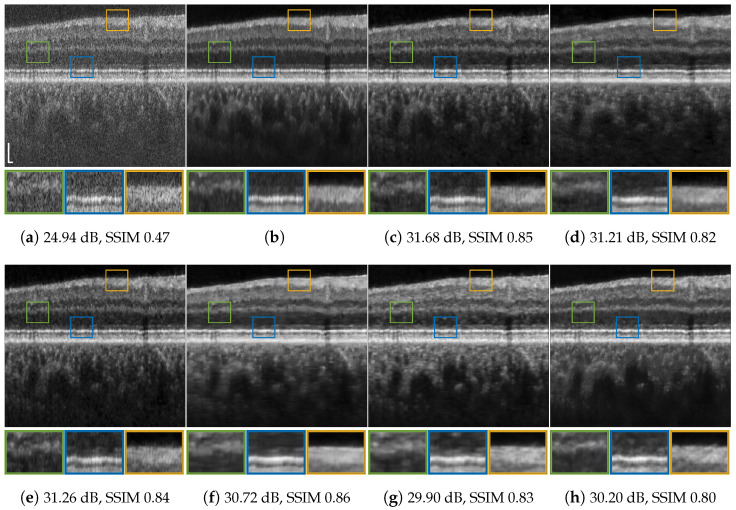
Retinal data speckle suppression: (**a**) cross-sectional human retina in vivo $p_{x}^{s}=2$; (**b**) despeckled (NLM) image used as the ground truth; (**c**) DRNN trained with 100 columns of retinal image $p_{x}^{s}=p_{x}^{t}=2$. System mismatch: (**d**) DRNN following lateral decimation of the target input by a factor of 2, $p_{x}^{t}=1$; (**e**) DRNN following lateral interpolating of the input, $p_{x}^{t}=3$. System and tissue mismatch: (**f**) RNN-GAN trained with 100 first columns of chicken muscle and blueberry, $p_{x}^{s}=3$; (**g**) RNN-GAN trained with 200 last columns of blueberry; (**h**) U-Net trained with blueberry image of size 256×256, $p_{x}^{s}=3$. Scale bars, 200 µm.

**Figure 5:**
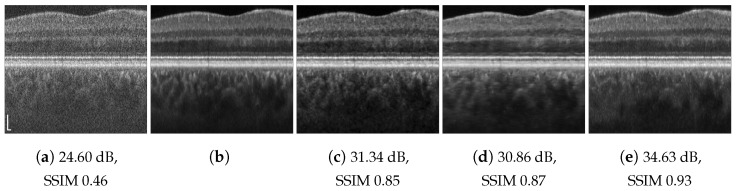
Retinal data speckle suppression: (**a**) cross-sectional human retina in vivo, $p_{x}^{t}=2$; (**b**) despeckled (NLM) image used as the ground truth; (**c**) DRNN trained with 100 columns of retinal image $p_{x}^{s}=p_{x}^{t}=2$; (**d**) RNN-GAN trained with 100 first columns of chicken muscle and blueberry, $p_{x}^{s}=3$; (**e**) U-Net trained with retinal image of size 256×256, $p_{x}^{s}=2$. Scale bars are 200 µm.

**Figure 6:**
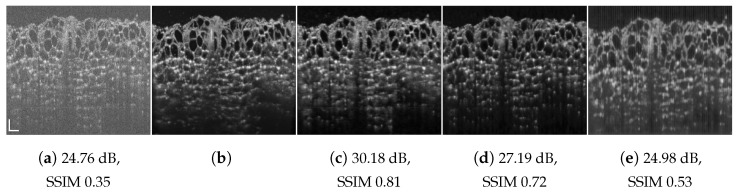
Blueberry speckle suppression results: (**a**) speckled acquired tomogram; (**b**) despeckled via angular compounding the used ground truth; (**c**) RNN-GAN trained with 200 last columns of blueberry, $p_{x}^{s}=p_{x}^{t}=3$; (**d**) DRNN trained with 100 columns of human retinal image, $p_{x}^{s}=2$; (**e**) U-Net trained with 256×256 chicken skin image, $p_{x}^{s}=2$. Scale bars are 200 µm.

**Figure 7:**
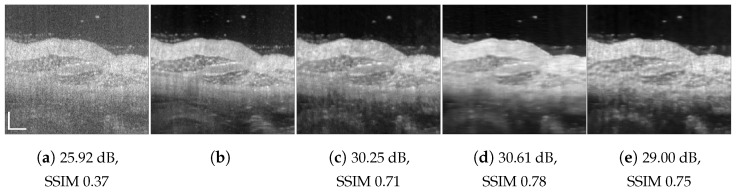
Chicken skin speckle suppression results: (**a**) speckled acquired tomogram; (**b**) AC ground truth, averaged over 60 tomograms; (**c**) DRNN trained with 100 columns of human retinal image, $p_{x}^{s}=p_{x}^{t}=2$; (**d**) RNN-GAN trained with 100 first columns of chicken muscle and blueberry, $p_{x}^{s}=3$; (**e**) RNN-GAN trained with 200 last columns of blueberry, $p_{x}^{s}=3$. Scale bars are 200 µm.

**Figure 8:**
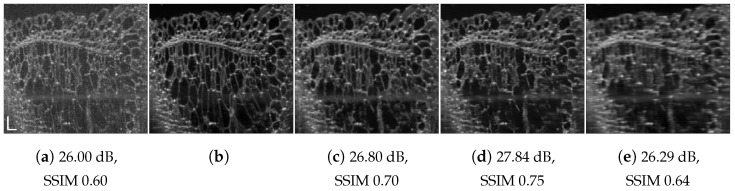
Cucumber speckle suppression results: (**a**) speckled acquired tomogram, $p_{x}^{t}=1$; (**b**) ground truth averaged over 301 tomograms; (**c**) DRNN trained with human retina image, $p_{x}^{s}=2$; (**d**) RNN-GAN trained with 200 columns of blueberry image decimated in the lateral direction by a factor of 8/3, $p_{x}^{s}=p_{x}^{t}=1$; (**e**) RNN-GAN trained with 200 columns of blueberry and chicken, $p_{x}^{s}=3$. Scale bars are 200 µm.

**Figure 9:**
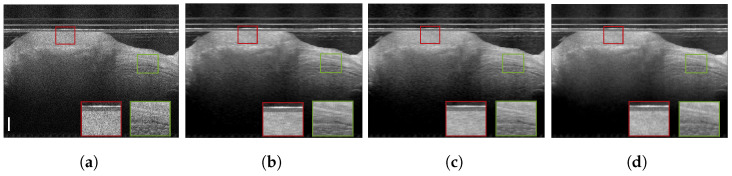
Cardiovascular-I speckle suppression results (in Cartesian coordinates): (**a**) speckled acquired tomogram, $p_{x}^{t}=2$; (**b**) DRNN trained with 100 columns of human retinal image, $p_{x}^{s}=2$; (**c**) RNN-GAN trained with 200 columns of blueberry and chicken images, $p_{x}^{s}=3$; (**d**) U-Net trained with retinal data image of size 448×256, $p_{x}^{s}=2$. Scale bar is 500 µm.

**Figure 10:**
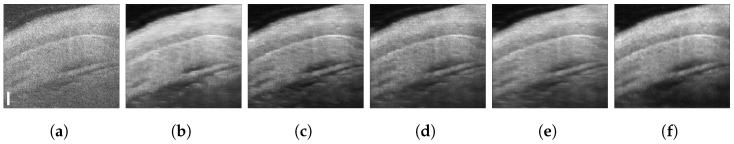
Cardiovascular-II speckle suppression results (in Cartesian coordinates): (**a**) cropped speckled acquired tomogram of size 371×311, $p_{x}^{t}=1$; (**b**) OCT-RNN trained with 100 first columns of chicken muscle, $p_{x}^{s}=3$; (**c**) DRNN trained with 100 columns of human retinal image, $p_{x}^{s}=2$; (**d**) RNN-GAN trained with decimated retinal data, $p_{x}^{s}=1$; (**e**) RNN-GAN trained with interpolated retinal data, $p_{x}^{s}=3$; (**f**) U-Net trained with retinal data image of size 448×256, $p_{x}^{s}=2$. Scale bar is 200 µm.

Figure 1, Figure 4, Figure 5, Figure 6, Figure 7, Figure 8, Figure 9 and Figure 10 depict the obtained despeckled predictions for the ex vivo samples, as well as for in vivo retinal data and intravascular OCT images, employing four methods: RNN, DRNN, RNN-GAN, and U-Net [32]. Please zoom-in on screen to see the differences. The U-Net has about $8.2\times 10^{6}$ parameters (eight-times the RNN’s number of parameters), and it trains with patches of size 64×64. Note that all four proposed methods were trained with either one example pair of the speckled image and its corresponding ground truth, a cropped part of an image, or very few examples. Visually observing the results in different scenarios, overall, the proposed approach efficiently suppresses speckle, while preserving and enhancing visual detailed structure.

To test the DRNN’s performance in different domains, we trained it with 100 columns of acquired in vivo human retinal cross-section, presented in Figure 4a. Figure 4b presents the ground truth obtained as described in [12]. As can be observed, the DRNN approach generalizes well, both with matching tissues and imaging systems, as well as in cases of tissue and system mismatch. The DRNN produces good visual quality and efficiently suppresses speckle, even without preprocessing domain adaptation. As we theoretically established, applying the source-trained system to a target with a lower lateral sampling resolution ratio indeed smooths the result (e.g., Figure 8e), whereas a target input with a higher lateral sampling resolution ratio results in a detailed structure with minor speckle residuals and (e.g., Figure 4e). Visually observing the other methods’ results leads to similar conclusions.

We quantitatively evaluated the proposed approaches by comparing the peak-signal-to-noise ratio (PSNR) and structural similarity index (SSIM) of their results with respect to the images assumed as ground truth. Table 2 compares the average PSNR and SSIM score for the above four one-shot learning methods with matching system and tissue. As can be seen, a significant increase in PSNR and SSIM scores is achieved for all methods. RNN-GAN and U-Net have the highest scores in most cases. U-Net usually yields the highest scores; yet, as can be observed in Figure 6e, it can produce unexpected visible artifacts in some cases. The U-Net has more capacity, therefore, it tends to memorize the training image better, but generalize worse.

Note that PSNR and SSIM scores not always reliably represent the perceptual quality or desired features of the images [51]. An image that minimizes the mean distance in any metric will necessarily suffer from a degradation in perceptual quality (the “perception–distortion” trade-off). Denoising is inherently an ill-posed problem. That is, a given input may have multiple correct solutions. The minimum-mean-squared error (MMSE) solutions are inclined to average these possible correct outcomes. In the presence of low SNRs, an averaging strategy often leads to output images with blurry edges and unclear fine details.

Keep in mind that AC-despeckled images are a result of averaging of numerous images, whereas our system’s predictions rely solely on a single observation; therefore, the reconstructions are notably more loyal to the single observed speckled image. Furthermore, although AC images are referred to as the ground truth, they may suffer from inaccuracies related to the stage tilting and its processing. The NLM ground truth also may suffer from residual speckle and blurring. As can seen in Figure 4 and Figure 5, the proposed models were able to remove some of these artifacts.

It is worth noting that, despite the growing interest in supervised learning methods for OCT despeckling, many competing (non few-shot learning) methods do not provide open access to training datasets and results. As most of these methods are trained with compounded data or NLMs and as the goal of this study is to explore few-shot learning and domain awareness, rather than to achieve state-of-the-art results, we directly compare our results with the assumed ground truth.

**Table 2:** Average PSNR/SSIM obtained for different methods and datasets with training and testing matching acquisition systems and tissue types. Average scores are over 100 tomograms of size 256×256.

Dataset	Input	RNN-OCT	DRNN	RNN-GAN	U-Net
**Retina**	24.87/0.46	33.60/ 0.87	30.46/0.82	32.24/0.86	33.66/0.89
**Chicken**	24.29/0.29	27.97/0.61	29.41/0.63	30.81/0.74	32.50/0.77
**Blueberry**	24.98/0.48	27.15/0.63	27.57/0.69	28.18/0.76	28.09/0.76
**Chicken Skin**	26.12/0.44	29.64/0.71	29.59/0.69	30.49/0.78	30.26/0.77
**Cucumber**	25.91/0.59	27.31/0.73	28.69/0.73	28.85/0.79	28.52/0.81

Table 3 provides quantitative scores for the proposed domain adaptation approach for various pairs of source and target, differing in acquisition system and tissue type, for RNN-GAN and U-Net. Notably, both approaches result in a significant increase in the PSNR and SSIM scores. Note that the images differ not only in their sampling resolution ratio, but also by the nature of the ground truth used for training. Namely, the AC images have a different texture and visual appearance than the NLMs. Regardless of the PSNR and SSIM scores, the trained model often tends to adopt the visual characteristics of the source data. This tendency may also be perceived as an advantage in the absence of a ground truth, as can be seen in Figure 4g. The observed speckled image may originate in many plausible reconstructions with varying textures and fine details and different semantic information [56]. The above results somewhat offer a user-dependent degree of freedom. Unfortunately, in our experiments, the domain randomization strategy [57] failed to generalize well.

**Table 3:** Domain-aware PSNR/SSIM obtained for different methods and datasets, with training and testing acquisition systems and tissue types mismatch, with preprocessing adapting the sampling resolution ratio. * denotes cases where domain adaption was not applied.

Target Data	Source Data	RNN-GAN	U-Net
Retina	Blueberry and Chicken	30.86/0.87	31.80/0.85
Chicken	Chicken Skin	31.33/0.69	31.08/0.68
Chicken	Retina	29.08/0.63	31.89/0.70
Blueberry	Retina	27.78/0.69	28.33/0.77
Chicken Skin	Blueberry and Chicken	30.68/0.76	30.43/0.71
Chicken Skin	Retina	31.51/0.76 *	30.88/0.77 *
Cucumber	Blueberry	27.84/0.75	27.61/0.72 *
Cucumber	Retina	28.71/0.77 *	28.11/0.73

*Training Time and Computational Resources*

The proposed model offers substantial training time efficiency. The number of epochs for the first training content loss stage is 5–12 epochs, depending on the analysis patch size, batch size, and training image size. The adversarial loss training stage takes about 10–30 epochs. The total time of training is ***5–25 seconds** on a laptop GPU*. Training without an adversarial stage normally takes about 12 seconds. As a rule of thumb, training for too long can cause over-fitting and blurry images. Early stopping is recommended to avoid the model from over-fitting the single image used for training. Training times were measured on a standard laptop workstation equipped with a 12th Gen Intel(R) Core(TM) i7-12800H 2.40 GHz with 32.0 GB RAM, NVIDIA RTX A2000 8 GB Laptop GPU. Training can also be performed on a CPU in a few minutes. Inference time is 110.5 ms per B-line. U-Net training is usually longer and takes about 5.76 minutes (for 16 epochs). As far as we know, our results are the state-of-the-art in terms of optimized real-time training with minimal available training data.

**Figure 11:**
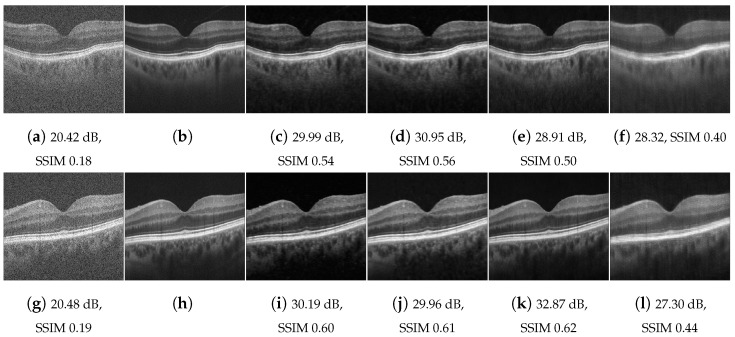
Retinal data speckle suppression: (**a**,**g**) cross-sectional human retina in vivo; (**b**,**h**) despeckled image (ground truth); (**c**,**i**) RNN trained with a (different) single retinal image; (**d**,**j**) RNN-GAN trained with 200 first columns the test image; (**e**,**k**) U-Net trained with a (different) single retinal image; (**f**,**l**) SM-GAN trained with 3900 example pairs.

Lastly, Figure 11 presents a visual comparison of our proposed one-shot RNN-GAN and U-Net results in comparison with SM-GAN [17], trained with 3900 example pairs of speckled and despeckled OCT images. The retinal SD-OCT and the corresponding ground truths are borrowed from the dataset in [58]. As can be seen, our approach is able to reduce speckle, while preserving perceptual quality and contrast. The comparison showcases the good generalization despite training only with a single image or part of an image.

## 6. Discussion and Conclusions

In this work, we analyzed the critical challenge of supervised learning domain adaptation for the task of OCT speckle suppression, with diverse imaging systems, given limited ground truth data. We focused on an RNN patch-based approach that is both flexible and efficient in terms of the patch size and number of parameters and less prone to low-frequency bias. We further designed a suitable adversarial loss training stage of relatively low complexity. We also demonstrated the applicability of our proposed point of view to a one-shot U-Net. Future research can potentially investigate other architectures. We observed that the proposed RNN-GAN evades cross-hatch pattern artifacts, which are oftentimes induced by the U-Net. Generative models are known to be able to produce high-quality samples in many domains. That said, in some cases, GANs’ training convergence can be highly sensitive to the optimization and architecture choices.

For the challenge of domain adaptation, we suggest a rather simplified point of view that can facilitate efficient deployment both for research and industrial purposes. Our future work will attempt to provide mathematical guarantees for the validity of possible resolution enhancement illuminated by the domain-aware learning perspective above. The efficiency of the proposed training is particularly interesting in applications with limited exposure time constraints and, as such, has the potential to substantially advance practical OCT implementations.

Our results challenge the assumption that training datasets must consist of a large representation of the entire high-dimensional input data probability distribution, thus evading the curse of dimensionality. The proposed few-shot learning framework may be related to the information theoretic asymptotic equipartition property on sample complexity [33,59]. Our results can inspire the design of novel few-shot learning systems for medical imaging and can also be of interest to the wider deep learning and signal-processing community. The models were formulated for two-dimensional (2D) signals, but can easily be adapted to other data dimensions. Future work can investigate the applicability of the proposed approach to other tasks and to cross-modality domain adaptation.

## Data Availability

Not applicable.

## Appendix A

**Proof** **of Theorem 1.** Let us assume source input–output pairs $\left\{y^{s},x^{s}\right\}\in R^{M\times N}$, with distribution $\left\{y^{s},x^{s}\right\}∼P_{S}\left(y^{s},x^{s}\right)$ in the source domain S, and samples $\left\{y^{t},x^{t}\right\}\in R^{M\times N}$ in the target domain T such that $\left\{y^{t},x^{t}\right\}∼P_{T}\left(y^{t},x^{t}\right)$. The predictor is trained and generalizes well, such that

(A1) $$L\left(F_{S}\right)=E_{\left(x,y\right)∼P_{S}}∥F_{S}\left(y^{s}\left[m,n\right]\right)-x^{s}\left[m,n\right]{∥}_{2}<\varepsilon ,$$

where $E_{\left(x,y\right)∼P_{X,Y}}\left(\cdot \right)$ denotes the expectation over $P_{X,Y}$ and ${∥\cdot ∥}_{2}$ denotes the $\ell_{2}$-norm, ${\varepsilon <<\min∥x∥}_{2}$. As mentioned above, in the OCT speckle-suppression problem, the coherent speckled input is

(A2) $$y^{s}\left[m,n\right]=|f^{s}\left[m,n\right]*\alpha^{s}\left[m,n\right]{|}^{2},$$

while the corresponding incoherent output is formulated as

(A3) $$x^{s}\left[m,n\right]=|f^{s}\left[m,n\right]{|}^{2}*|\alpha^{s}\left[m,n\right]{|}^{2}.$$

Similarly, the target input is

(A4) $$y^{t}\left[m,n\right]=|f^{t}\left[m,n\right]*\alpha^{t}\left[m,n\right]{|}^{2},$$

and the desired despeckled image in the target domain is

(A5) $$x^{t}\left[m,n\right]=|f^{t}\left[m,n\right]{|}^{2}*|\alpha^{t}\left[m,n\right]{|}^{2}.$$

We omit the scaled logarithm for simplicity. Applying the trained source system to the source data, we can assume $\forall y^{s}\left[m,n\right]\in R^{M\times N}\exists x^{s}\left[m,n\right]:x^{s}\left[m,n\right]\approx F_{S}\left(y^{s}\left[m,n\right]\right)$; therefore, applying the trained system to the target output, we have

(A6) $$F_{S}\left(y^{t}\left[m,n\right]\right)=x^{s}\left[m,n\right]{|}_{y^{s}\left[m,n\right]=y^{t}\left[m,n\right]}$$

In other words, the system’s output is its prediction for

(A7) $$y^{s}\left[m,n\right]=|f^{s}\left[m,n\right]*\alpha^{s}\left[m,n\right]{|}^{2}=|f^{t}\left[m,n\right]*\alpha^{t}\left[m,n\right]{|}^{2}=y^{t}\left[m,n\right]$$

where $\alpha^{t}\left[m,n\right]=\alpha_{z}^{t}\left[m\right]*\alpha_{x}^{t}\left[n\right]$ and $\alpha^{s}\left[m,n\right]=\alpha_{z}^{s}\left[m\right]*\alpha_{x}^{s}\left[n\right]$ are separable PSFs. The predictor does not “know” that its input does not belong to the same domain, and it would output the corresponding prediction in the source domain. Note that, in many applications, the target input often largely differs from the source input; yet, (A7) is justified by a patched-based learning strategy. For the sake of the proof’s outline, let us assume the target and source domains share an equivalent sampling resolution ratio in the axial direction, that is $\alpha_{z}^{s}\left[m\right]=\alpha_{z}^{t}\left[m\right]$. Let us examine two possible scenarios. (1) If $p_{x}^{s}<p_{x}^{t}$, there exist $\alpha_{x}^{s\to t}\left[n\right]$ such that $\alpha_{x}^{t}\left[n\right]=\alpha_{x}^{s}\left[n\right]*\alpha_{x}^{s\to t}\left[n\right]$. Then, (A7) is satisfied if

$$\begin{array}{cc} \left|f^{t}\left[m,n\right]*\alpha^{t}\left[m,n\right]\right| & =|f^{t}\left[m,n\right]*\alpha^{s\to t}\left[m,n\right]*\alpha^{s}\left[m,n\right]|\\ & =|f^{s}\left[m,n\right]*\alpha^{s}\left[m,n\right]|. \end{array}$$

Therefore,

(A8) $$\begin{array}{cc} F_{S}\left(y^{t}\left[m,n\right]\right) & =x^{s}\left[m,n\right]{|}_{y^{t}\left[m,n\right]=y^{s}\left[m,n\right]}\\ & =|f^{t}\left[m,n\right]*\alpha^{s\to t}\left[m,n\right]{|}^{2}*|\alpha^{s}\left[m,n\right]{|}^{2}. \end{array}$$

In other words, the output resolution is determined by the source resolution $\alpha^{s}\left[m,n\right]$. The tomogram component $|f^{t}\left[m,n\right]*\alpha^{s\to t}\left[m,n\right]|$ is a lower-resolution version of the desired target tomogram $|f^{t}\left[m,n\right]|$. (2) If $p_{x}^{s}>p_{x}^{t}$, there exist $\alpha_{x}^{t\to s}\left[n\right]$ such that $\alpha_{x}^{s}\left[n\right]=\alpha_{x}^{t}\left[n\right]*\alpha_{x}^{t\to s}\left[n\right]$. Then, (A7) is satisfied if

$$\begin{array}{cc} \left|f^{t}\left[m,n\right]*\alpha^{t}\left[m,n\right]\right| & =|f^{s}\left[m,n\right]*\alpha^{s}\left[m,n\right]|\\ & =|f^{s}\left[m,n\right]*\alpha^{t\to s}\left[m,n\right]*\alpha^{t}\left[m,n\right]| \end{array}$$

Thus,

(A9) $$\begin{array}{cc} & F_{S}\left(y^{t}\left[m,n\right]\right)=x^{s}\left[m,n\right]{|}_{y^{s}\left[m,n\right]=y^{t}\left[m,n\right]}\\ & =|f^{s}\left[m,n\right]{|}^{2}*|\alpha^{s}\left[m,n\right]{|}^{2}{|}_{f^{t}\left[m,n\right]=f^{s}\left[m,n\right]*\alpha^{t\to s}\left[m,n\right]}. \end{array}$$

In other words, the resolution of the output will be determined by the source resolution $\alpha^{s}\left[m,n\right]$. Furthermore, since the solution obeys $f^{t}\left[m,n\right]=f^{s}\left[m,n\right]*\alpha^{t\to s}\left[m,n\right]$, the tomogram component $|f^{s}\left[m,n\right]|$ is, in a sense, a speckled super-resolved version of the target tomogram $|f^{t}\left[m,n\right]|$. Note that (A9) is true under the assumption that $\forall f^{t}\left[m,n\right]\exists f^{s}\left[m,n\right]:f^{t}\left[m,n\right]=f^{s}\left[m,n\right]*\alpha^{t\to s}\left[m,n\right]$. In other words, $f^{s}\left[m,n\right]$ is a super-resolved version of $f^{t}\left[m,n\right]$, which is safe to assume since the resolution of f[m,n] is well above the size of point scatters’ particles. Specifically, for a Gaussian PSF, we know that the convolution of two Gaussian with mean $\mu_{1},\mu_{2}$ and variance $\sigma_{1}^{2}$, $\sigma_{2}^{2}$ is a Gaussian with mean $\mu =\mu_{1}+\mu_{2}$ and variance $\sigma^{2}=\sigma_{1}^{2}+\sigma_{2}^{2}$. The same proof applies to the cases where $p_{z}^{s}≶p_{z}^{t}$ are interchangeable or a combination of $p_{z},p_{x}$ due to separability. For the general case, we can write

(A10) $$\alpha^{s}\left[m,n\right]*\alpha^{s\to t}\left[m,n\right]=\alpha^{t}\left[m,n\right]*\alpha^{t\to s}\left[m,n\right].$$

Therefore,

(A11) $$\begin{array}{cc} & F_{S}\left(y^{t}\left[m,n\right]\right)=x^{s}\left[m,n\right]{|}_{y^{s}\left[m,n\right]=y^{t}\left[m,n\right]}=\\ & |f^{s}\left[m,n\right]{|}^{2}*|\alpha^{s}\left[m,n\right]{|}^{2}{|}_{f^{t}\left[m,n\right]*\alpha^{s\to t}\left[m,n\right]=f^{s}\left[m,n\right]*\alpha^{t\to s}\left[m,n\right]}. \end{array}$$

For example, when $p_{x}^{s}<p_{x}^{t}$ and $p_{z}^{s}>p_{z}^{t}$, ${\hat{x}}^{t}=F_{S}\left\{y^{t}\right\}$, we have

$$F_{S}\left(y^{t}\right)=|f^{s}\left[m,n\right]{|}^{2}*|\alpha^{s}\left[m,n\right]{|}^{2}{|}_{f^{t}\left[m,n\right]*\alpha_{x}^{s\to t}\left[n\right]=f^{s}\left[m,n\right]*\alpha_{z}^{t\to s}\left[m\right]}.$$

 □
